# Effect of Tea Saponins on Rumen Microbiota and Rumen Function in Qinchuan Beef Cattle

**DOI:** 10.3390/microorganisms11020374

**Published:** 2023-02-01

**Authors:** Xiaopeng Qu, Sayed Haidar Abbas Raza, Yanqing Zhao, Jiahan Deng, Jing Ma, Juze Wang, Nada Alkhorayef, Samia S. Alkhalil, Sameer D. Pant, Hongtao Lei, Linsen Zan

**Affiliations:** 1 College of Animal Science and Technology, Northwest A&F University, Yangling, Xianyang 712100, China; 2 Safety of Livestock and Poultry Products, College of Food Science, South China Agricultural University, Guangzhou 510642, China; 3 Department of Clinical Laboratory Science, College of Applied Medical Sciences, Al-Quway’iyah Shaqra University, Al-Quway’iyah 13343, Saudi Arabia; 4 Department of Clinical Laboratory Sciences, Faculty of Applied Medical Sciences, Shaqra University, Shaqra 11961, Saudi Arabia; 5 Gulbali Institute, Charles Sturt University, Boorooma Street, Wagga Wagga, NSW 2678, Australia; 6 National Beef Cattle Improvement Center, Yangling, Xianyang 712100, China

**Keywords:** cattle, feed additives, rumen, microflora, saponins

## Abstract

Antibiotics can promote livestock growth but have side effects, so the search for safe and effective alternatives to antibiotics is urgent. This study aimed to evaluate the effect of supplementing cattle feed with tea saponins on ruminal bacteria and fungi. Sixteen Qinchuan beef cattle with a live body weight of 250 ± 10 kg were divided into four groups (four animals in each group) using a completely randomized experimental design. Four different levels of tea saponins were provided to the Qinchuan cattle as treatments, including 0 g/cattle per day control, CON), 10 g/cattle per day (low-level, LT), 20 g/cattle per day (medium-level, MT) and 30 g/cattle per day (high-level, HT). The pre-feeding period was 10 days and the official period was 80 days in this experiment. After 90 days of feeding, the rumen fluid from sixteen Qinchuan beef cattle was collected using an oral stomach tube for evaluating changes in ruminal microbiota and rumen fermentation parameters. Results indicate that the total VFAs and proportions of propionate in the LT group was significantly higher than that in the CON and HT groups (*p* < 0.05). For ruminal bacteria, results indicate that the Chao1 index of the MT group was significantly lower than the CON and HT groups (*p* < 0.05). The phyla Bacteroidetes and Firmicutes were found to be the most abundant in all treatment groups, with the LT group having significantly increased relative abundances of Proteobacteria, Actinobacteria and Ascomycota at the phylum level (*p* < 0.05). The relative abundance of Bacteroides was found to be relatively lower in the LT, MT and HT treatment groups compared with the CON treatment group at the genus level (*p* < 0.05). For ruminal fungi, the LT treatment group was found to have higher relative abundances of Saccharomyces and Aspergillus, and lower relative abundances of Succiniclasticum and Bacteroides at the at the phylum level (*p* < 0.05). Compared with the CON treatment group, a significant increase in the relative abundance of Saccharomyces and Aspergillus were observed in the LT treatment group at the genus level (*p* < 0.05). PICRUSt analyses identified pathways associated with Xenobiotic biodegradation and metabolism and glycolysisIII to be significantly enriched in the LT and HT treatment groups (*p* < 0.05). These findings could provide insights on how tea saponins may influence ruminal bacteria and fungi, providing a theoretical basis for replacing antibiotics with tea saponins for promoting growth in cattle.

## 1. Introduction

Adding antibiotics to animal feed can improve livestock production. However, the use of antibiotics is controversial as it can lead to the emergence of antibiotic-resistant strains that pose a risk to both human and livestock health [1]. In addition, the use of antibiotics is prohibited in some regions of the world, such as the European Union. Therefore, antibiotic alternatives need to be developed to improve livestock productivity. In this context, recent studies indicate that secondary metabolites such as tannins, saponins, flavonoids and essential oils have the potential to regulate digestive tract metabolism and improve animal production efficiency [2,3]. Compared with antibiotics, the use of secondary metabolites is advantageous as they are safe and effective, without hormonal consequences or other negative side effects [4].

Saponins are widely distributed in ginseng, alfalfa, tea and other plants, and their roles can vary based on their sources [5]. Tea saponins are triterpenoids widely distributed in the roots, stems, leaves and seeds of the genus *Camellia* [6]. Previous studies indicate that saponins have the potential to both increase feed digestibility, and reduce methane emissions in ruminants. For example, saponin supplementation in sheep appears to increase digestibility of organic matter, neutral detergent fibre and acid detergent fibre by 9.6%, 27.9% and 38%, respectively [7]. Furthermore, in an in vitro fermentation study, addition to saponins led to a 32.5% reduction in methane production, coupled with significantly higher short chain fatty acid and metabolizable energy production [8]. Overall, these studies support a potential role for tea saponins in regulating nutrient digestion.

Rumen is an important digestive organ in livestock, which houses a complex ecosystem composed of a large number and a variety of microbial species, primarily including bacteria, protozoa and fungi [9]. These microbial species play a crucial role in the digestive physiology of ruminants, and therefore it is plausible that the effect of saponins on ruminant digestion is mediated by modulating rumen microbiota. In fact, recent studies already provide evidence that tea saponins improve rumen fermentation by killing protozoa [10]. However, apart from protozoa, tea saponins may also modulate rumen metabolism by influencing ruminal bacteria and fungi [10,11]. Moreover, tea saponins may potentially cause toxicity when added to feed at high levels, and at least part of these toxic effects may be mediated via ruminal microbiota. To the best of our knowledge, previous microbiome-based studies have not characterized the effects of tea saponins on rumen bacteria and fungi. Therefore, this study aims to characterize the effect of feeding different levels of tea saponins on rumen microbiota and rumen function in Qinchuan cattle. The broader aim of this study is to provide a theoretical basis for the use of tea saponin in ruminants.

## 2. Materials and Methods

### 2.1. Ethics Statement

The procedures for animal handling for experiments were approved by the Committee of Experimental Animal Management at Northwest Agriculture and Forestry University, China (protocol number: NWAFUCAST2018-72110). Moreover, all applicable rules and regulation of the organization and government were followed regarding the ethical use of experimental animals.

### 2.2. Animals, Experimental Design and Diets

The Qinchuan cattle selected for this study were provided by the breeding farm of the National Beef Cattle Improvement Center (Yangling, Xianyang, China). Tea saponins were extracted from camellia seeds (Camellia-Oilfera Abel, 85–90% purity of triterpenoid saponins) and were provided by Xi’an Green Biotechnology (Xi’an, China). A completely randomized experimental design was adopted. Sixteen 14-month-old Qinchuan cattle (female) with good body condition and weight (an average of 250 ± 10 kg) were selected and randomly divided into 4 groups with no significant difference in weight. Four different levels of tea saponins were provided to Qinchuan cattle as treatments, including 0 g/cattle per day (control, CON), 10 g/cattle per day (low-level, LT), 20 g/cattle per day (medium-level, MT) and 30 g/cattle per day (high-level, HT), and tea saponins are evenly mixed in the diet of each cattle. Each treatment group comprised 4 cattle. The pre-feeding period was 10 days, and the trial period was 80 days in this experiment. All cattle were fed twice daily at 08:30 and 15:30 hrs, and they were placed in individual pens (2.5 m × 1.8 m), clean, fresh water and the total mixed ration were provided ad libitum. Ingredients and nutritional compositions of the experimental diets were presented in Table 1.

### 2.3. Sample Collection and Processing

The collection of ruminal fluid was performed 2 h after the morning feeding on day 90 of treatment. Rumen fluid samples were collected through the oral cavity using a rumen tube. The initial 150 mL of ruminal fluid was discarded to avoid contamination with saliva, and the subsequent 100 mL was retained. The pH was measured immediately after collection, and subsequently, the sample was equally divided in two aliquots. The first aliquot was placed in a 50 mL cryotube and stored at −80 °C for the determination of rumen microbiota, and the other aliquot was used for the determination of fatty acids (VFAs). VFA concentrations were determined using gas chromatography (GC-14B; Shimadzu Corp., Kyoto, Japan) as per previously published protocols [12].

### 2.4. DNA Extraction and Illumina Sequencing

Genomic DNA extraction was performed using CTAB method, and extracted DNA concentration and purity was assessed via 1% agarose gel electrophoresis. The hypervariable V3-V4 region in the bacterial 16S rRNA of all samples were targeted for PCR amplification using the bacterial universal primers 341F (5′-CCTAYGGGRBGCASCAG-3′) and 806R (5′-GGACTACNNGGGTATCTAAT-3′) [13]. The hypervariable V4 region in the fungal 18S rRNA of all samples were targeted for PCR amplification using the fungal universal primers528F(5′-GCGGTAATTCCAGCTCCAA-3′) and 706R (5′-AATCCAAGAATTTCACCTCT-3′) [14]. All PCR reactions were carried out with 15 µL of Phusion^®^ High-Fidelity PCR Master Mix (New England Biolabs, Ipswich, MA, USA); 2 µM of forward and reverse primers, and about 10 ng template DNA. Thermal cycling consisted of initial denaturation at 98 °C for 1 min, followed by 30 cycles of denaturation at 98 °C for 10 s, annealing at 50 °C for 30 s and elongation at 72 °C for 30 s. Finally, 72 °C for 5 min.

Mix same volume of 1XTAE buffer with PCR products and operate electrophoresis on 2% agarose gel for detection. PCR products was mixed in equidensity ratios. Then, mixture PCR products was purified with Qiagen Gel Extraction Kit (Qiagen, Germany). Sequencing libraries were generated using TruSeq^®^ DNA PCR-Free Sample Preparation Kit (Illumina, San Diego, CA, USA) following manufacturer’s recommendations and index codes were added. The library quality was assessed on the Qubit@ 2.0 Fluorometer (Thermo Scientific, Waltham, MA, USA). At last, the library was sequenced on an Illumina NovaSeq platform.

### 2.5. Bioinformatics Analysis

Raw data in the form of FASTQ files were imported into QIIME2 using ‘qiime tools import’ program. Demultiplexed sequences from each sample were quality filtered and trimmed, de-noised, merged and then the chimeric sequences were identified and removed using the QIIME2 dada2 plugin to obtain the feature table of amplicon sequence variant (ASV) [15]. The QIIME2 feature-classifier plugin was then used to align ASV sequences to a pre-trained SILVA 99% database to generate the taxonomy table [16]. Diversity metrics were calculated using the core-diversity plugin within QIIME2. Feature level alpha diversity indices, such as Simpson diversity index, Chao1 richness estimator, Shannon diversity index and Faith’s phylogenetics diversity index were calculated to estimate the microbial diversity within an individual sample. The alpha diversity indices were analysed using a completely randomized design by one-way analysis of variance. PLS-DA (Partial least squares discriminant analysis) was introduced as a supervised model to reveal beta diversity, using the “plsda” function in R package “mixOmics” [17]. Significance of the microbial differences at the phylum and genus level were identified using a Kruskal Wallis test. The primary differentially abundant genera were analysed by the linear discrimination analysis (LDA) coupled with the effect size (LEfSe) method [18]. The bioinformatic tool PICRUSt was used to predict metabolic genes based on the 16S rRNA data of bacteria and 18S rRNA data of fungi. Metabolic genes of bacteria were matched into metabolic pathways using Kyoto Encyclopedia of Genes and Genomes (KEGG), and metabolic genes of fungi were matched into metabolic pathways using Metabolic Pathways From all Domains of Life (MateCyc). Metabolic pathways with a *p*-value < 0.05 were considered statistically significant in terms of enrichment.

### 2.6. Statistical Analysis

Statistical analysis of rumen fermentation parameter data was performed using the MIXED procedure in SAS (ver. 9.4; SAS Institute, Cary, NC, USA), using a statistical model that included the fixed effects of weight and diet, and the random effect associated with individual animals. The Tukey’s test was used to compare the differences among the 4 treatment groups. Orthogonal polynomial contrasts were used to analyze the linear and quadratic effects of the tea saponin levels. Significant differences in microbiota profiles at the phylum and genus level were identified using a Kruskal Wallis test. The alpha diversity indices and metabolic pathway were analysed using a completely randomized design by one-way analysis of variance. The statistical differences were represented as different letters, when the *p*-value < 0.05.

### 2.7. Data Accession Number

The raw sequencing data (Illumina) used in this study were deposited in the NCBI database, and is publicly available under the accession nos. PRJNA880688 and PRJNA880642.

## 3. Results

### 3.1. Impact of Tea Saponins Addition on the Diversity of Rumen Fermentation Parameters

The rumen fermentation parameters are listed in Table 2. The addition of tea saponin in feed does not affect pH, proportions of acetate, butyrate, iso-butyrate, valerate and iso-valerate. The total VFAs and proportions of propionate in the LT group was significantly higher than that in the CON and HT groups (*p* < 0.05). The acetate to propionate (A:P) ratio in the LT group was significantly lower than that in the CON and HT groups (*p* < 0.05). Further, as the tea saponin dosage increased, the proportion of butyrate increased linearly (*p* < 0.05).

### 3.2. Impact of Tea Saponins Addition on the Diversity of Cattle Ruminal Microbiota Diversity Analysis

Alpha diversity index analysis was performed on the four treatment groups of CON, LT, MT and HT. For ruminal bacteria, the results showed that the Chao1 index of the MT treatment group was significantly lower than the CON, LT and HT treatment groups, and the faith pd index of the MT treatment group was significantly lower than CON and LT treatment groups (*p* < 0.05). The four treatment groups showed no significant differences in Shannon index and Simpson indices (Table 3). These results indicated that the diversity of bacterial communities in CON, LT and HT treatment groups was significantly higher than that in the MT treatment group. For ruminal fungi, no significant differences were identified, and the four groups showed no significant difference in chao1, faith pd, Shannon and Simpson indices (Table 4).

**Figure 1:**
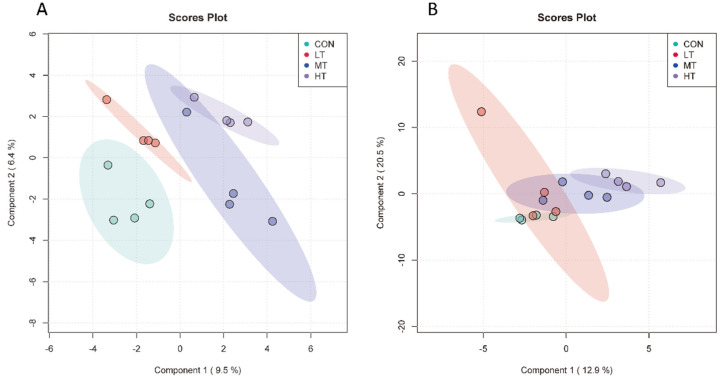
Partial least squares discriminant analysis of ruminal bacteria (**A**) and ruminal fungi (**B**) in different treatment groups.

Beta diversities of microbial communities of four groups were calculated and visualized by PLS-DA using Unweighted Unifrac distances. For ruminal bacteria, results showed that bacterial communities were distinct between the four treatment groups (Figure 1A). However, CON, LT, MT and HT groups overlapped with each other for ruminal fungi, indicating a greater degree of similarly fungal community structures among the four treatment groups (Figure 1B).

### 3.3. Analysis of Microbial Composition and Community Structure

**Figure 2:**
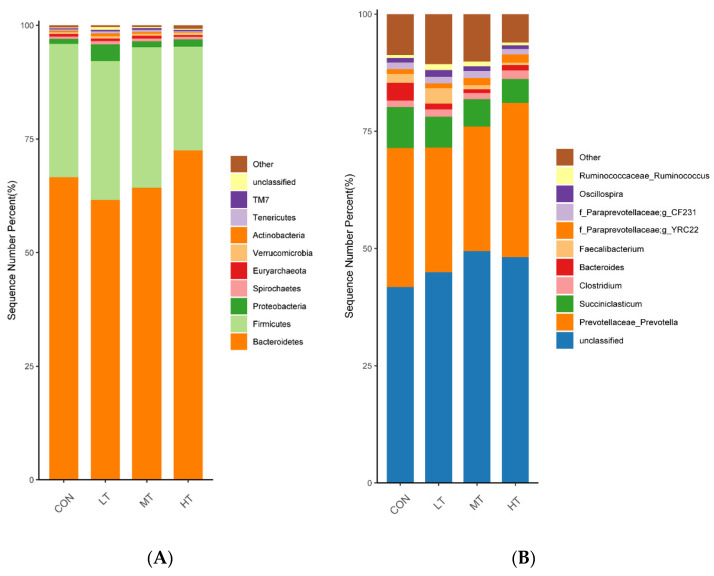
Distribution of bacterial taxa averaged under phyla (**A**) and genera (**B**) level across the different treatment groups (as a percentage of the total sequence).

In terms of relative abundances, the top 10 bacterial phyla are shown in Figure 2A, and the top 10 bacterial genera are shown in Figure 2B. At the phylum level (Figure 2A), the phyla Bacteroidetes and Firmicutes were found to have the highest relative abundances in all treatment groups. The relative abundance of Proteobacteria in the HT treatment group was significantly higher than that in the CON and MT treatment groups (*p* < 0.05). Similarly, the relative abundance of Actinobacteria in the LT treatment group was significantly higher than that in the CON, MT and HT treatment groups (*p* < 0.05). None of the other phyla was found to have significant differences in relative abundance within the four treatment groups (*p* > 0.05) (Table 5). At the genus level (Figure 2B), Prevotellaceae_Prevotella, Succiniclasticum, Clostridium and Bacteroides were found to have relatively high abundances in all treatment groups. As for genus-specific differences, a significant decrease in the relative abundance of Succiniclasticum, was observed in the MT and HT treatment groups compared with the CON treatment groups (*p* < 0.05). In addition, a lower relative abundance of Bacteroides was observed in the LT, MT and HT treatment groups compared with the CON treatment group (*p* < 0.05). The other genera had no significant differences between the four treatment groups (*p* > 0.05) (Table 5).

**Figure 3:**
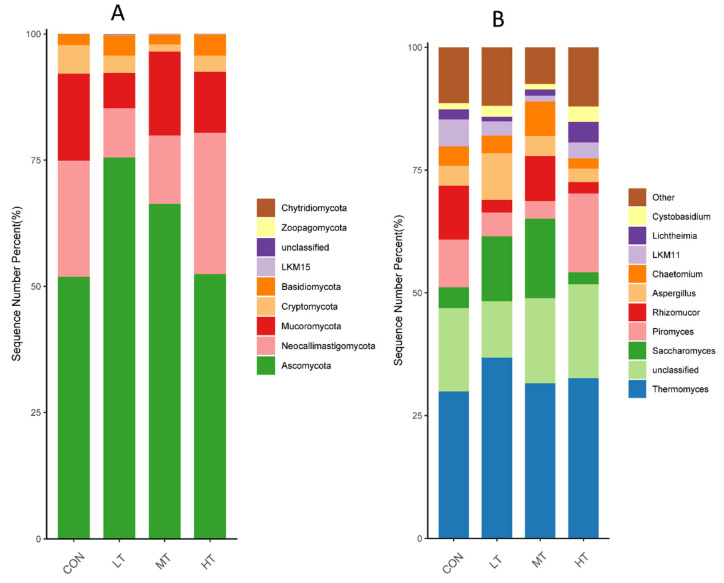
Distribution of fungal taxa averaged under phyla (**A**) and genera (**B**) level across the different treatment groups (as a percentage of the total sequence).

Rumen whose phylum-level relative abundance was in the top 10 of all the fungal communities is shown in Figure 3A. The genus of the top 10 is given in Figure 3B.

At the phylum level (Figure 3A), Ascomycota, Neocallimastigomycota, Mucoromycota, Cryptomycota and Basidiomycota occupied dominant positions for all groups. We found that the LT group had a significantly higher relative abundance of Ascomycota than did the CON and HT groups. (*p* < 0.05) (Table 6).

At the genus level (Figure 3B), Thermomyces, Saccharomyces, Piromyces, Rhizomucor and Aspergillus occupied dominant positions for all groups. Compared with the CON group, a significant increase in the relative abundance of Saccharomyces and Aspergillus were observed in the LT group (*p* < 0.05) (Table 5). Moreover, a lower relative abundance of Chaetomium was observed in the MT group compared with the MT group (*p* < 0.05).

### 3.4. Microbial Community Differences between Four Groups

**Figure 4:**
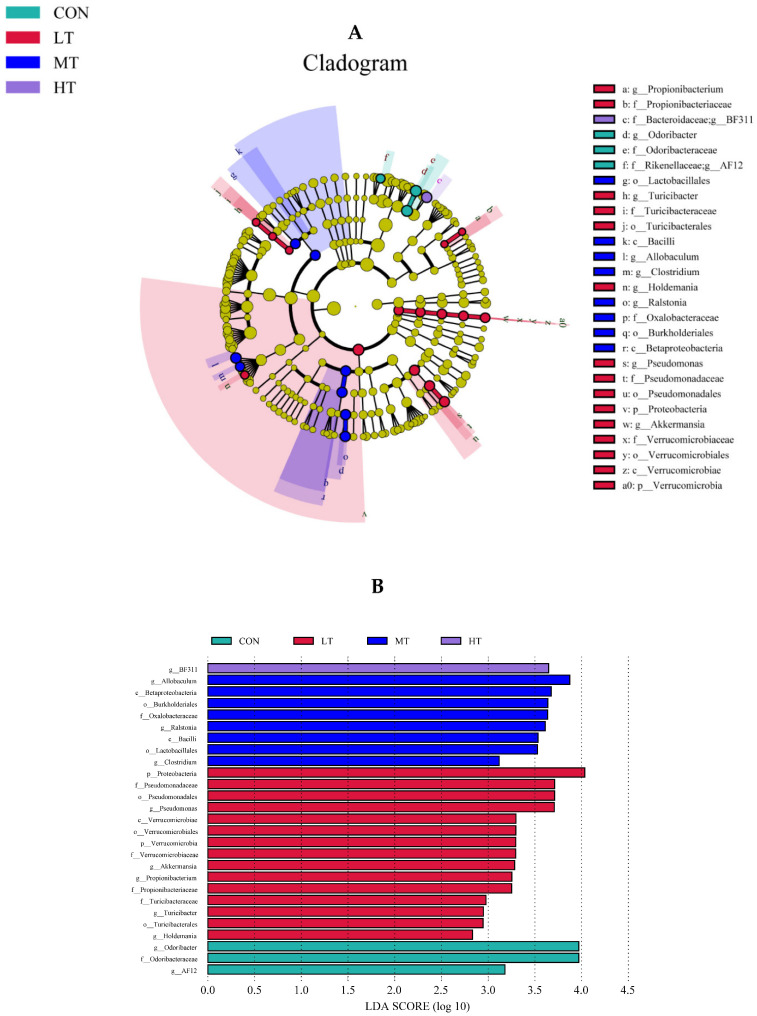
(**A**) LEfSe (linear discriminant analysis Effect Size) cladogram comparing bacterial communities among the four groups. (**B**) Histogram of LDA score calculated for each taxon ranging from phylum to genus among the four groups.

**Figure 5:**
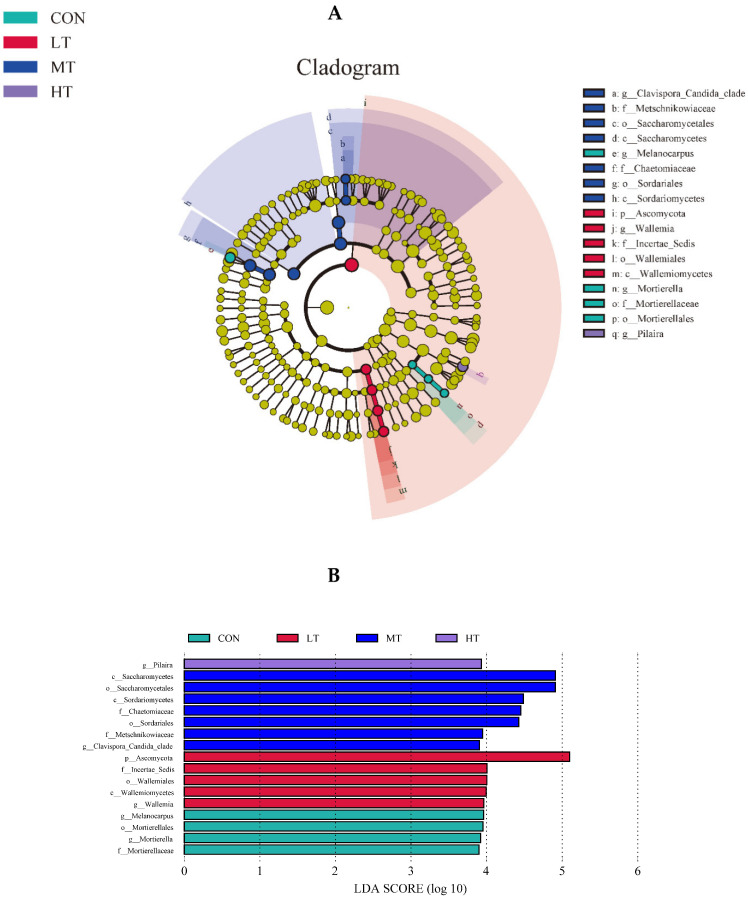
(**A**) LEfSe (linear discriminant analysis Effect Size) cladogram comparing fungal communities among the four groups. (**B**) Histogram of LDA score calculated for each taxon ranging from phylum to genus among the four groups.

The LefSe analysis was performed to identify microbial species that were differentially abundant between the treatment groups. For ruminal bacteria, data indicate that the most abundant clades in CON, LT, MT and HT treatment groups were 3, 15, 8 and 1, respectively (Figure 4A). Amongst them, the differentially abundant bacterial genus in the CON treatment group was Odoribacter, and the differentially abundant bacterial genus in the LT treatment group were Holdemania, Turicibacter, Propionibacterium, Akkermansia and Pseudomonas. The differentially abundant bacterial genus in MT was Ralstoniac and Clostridium, and BF311 was the differentially abundant bacterial genus in HC (Figure 4B). For ruminal fungi, results showed that the more abundant clades inCON, LT, MT and HT groups were 4, 5, 7 and 11, respectively (Figure 5A). Among these, the differentially abundant fungal genera in the CON treatment group were Mortierella and Melanocarpus, and in the LT treatment group was Wallemia. The Clavispora _Candida_ clade was the most differentially abundant fungal genus in the MT treatment group, and Pilaira was the most differentially abundant fungal genus in the HT treatment group (Figure 5B).

### 3.5. Functions Estimation

**Figure 6:**
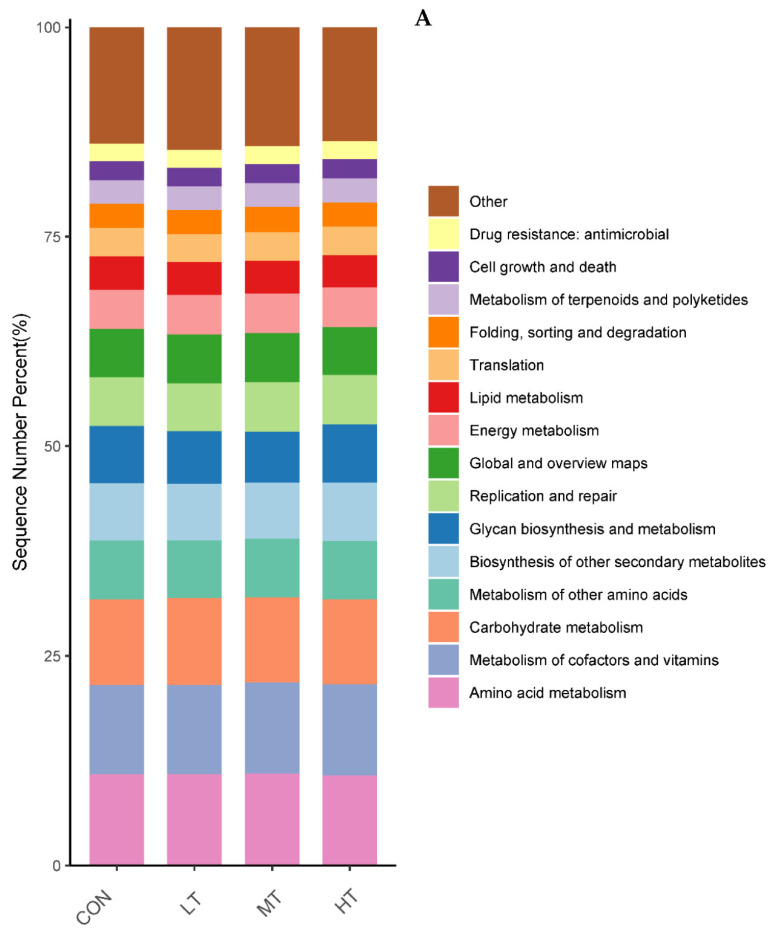

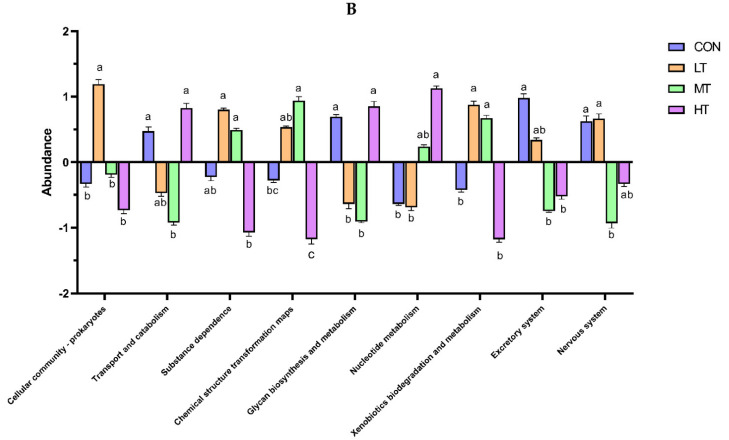
(**A**) Prediction of relative abundance of ruminal bacteria metabolic functions by Level 2 Kyoto Encyclopedia of Genes and Genomes (KEGG). (**B**) Functional differences of ruminal bacteria in four groups at KEGG-pathway level 2. The different letters are significantly different (*p* < 0.05).

**Figure 7:**
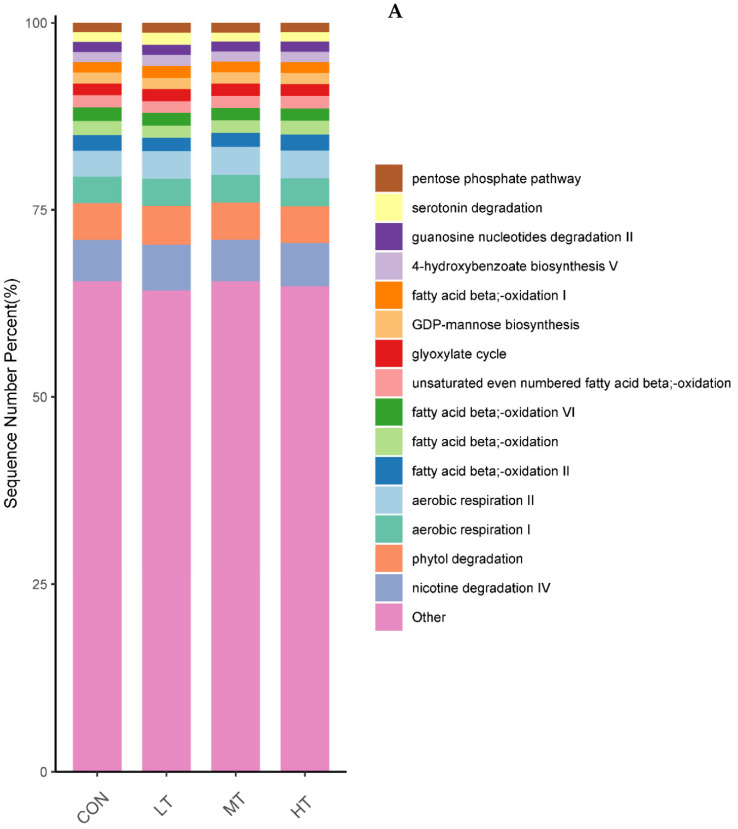

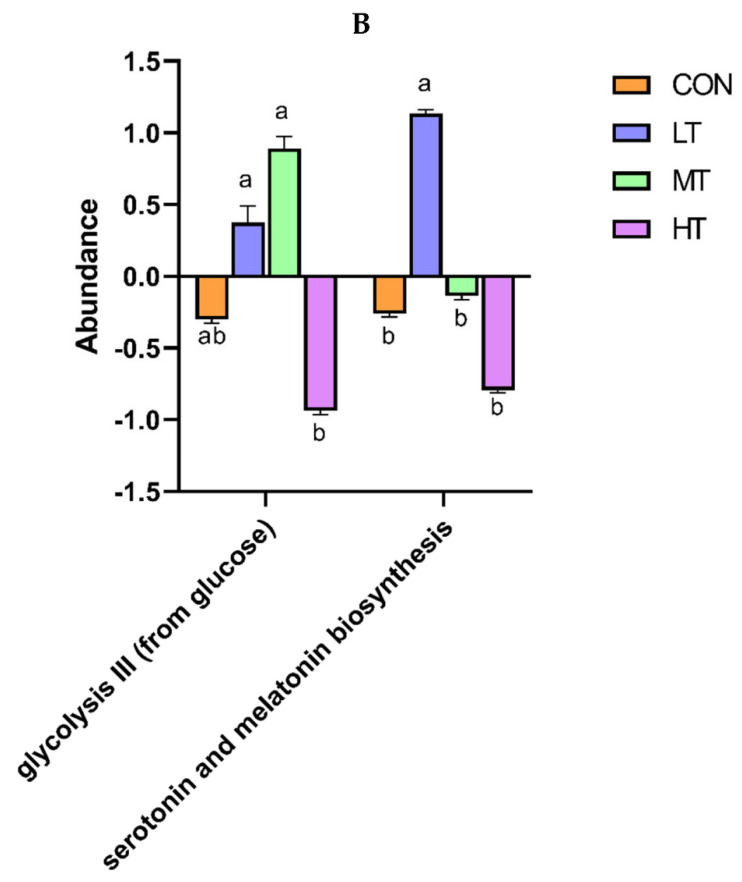
(**A**) Prediction of relative abundance of ruminal fungi metabolic functions by Metabolic Pathways From all Domains of Life (MateCyc). (**B**) Functional differences of ruminal fungi in four groups at MateCyc. The different letters are significantly different (*p* < 0.05).

PICRUSt was used to predict the function of microbial communities in the rumen of cattle. We identified 46 functional categories (KEGG-pathway level 2) associated with ruminal bacteria, the top 15 of which are noted in Figure 6A. A majority of these functional categories were found to be related with ‘metabolism’, such as Amino acid metabolism, Metabolism of cofactors and vitamins, Carbohydrate metabolism, Glycan biosynthesis and metabolism, Energy metabolism and Metabolism of terpenoids and polyketides. Functional categories with significant differences in enrichment between the treatment groups are shown in Figure 6B. We identified nine functional categories with significant differences, among which Xenobiotics biodegradation and metabolism functional genes were enriched in LT and MT groups (*p* < 0.05). Similarly, we identified 128 predictive functional categories associated with ruminal fungi curated in MateCyc-pathway database. The top 15 of these MateCyc-pathway functional categories are given in Figure 7A. These pathways involve carbohydrate biosynthesis, fatty acid oxidation and metabolites degradation. The functional categories with significant differences in enrichment between treatment groups are shown in Figure 7B, amongst which glycolysisIII (from glucose) functional genes were enriched in LT and MT groups (*p* < 0.05).

## 4. Discussion

Acetate, propionate and butyric acid constitute some of the main VFAs found in rumen, which are derived from the fermentation of carbohydrates in feed [19]. In this study, increasing levels of tea saponin supplementation did not significantly alter the content of acetate, butyrate, iso-butyrate, valerate and iso-valerate fatty acids. However, feed supplementation with low levels of tea saponin significantly increased total VFA content and the proportion of propionate fatty acid. These changes are likely attributable to alterations in the relative abundance of microbial species involved in carbohydrate decomposition, observed in the LT treatment group, such as Actinobacteria, Saccharomyces and Aspergillus. Similar studies in the past have also reported increased proportion of propionate, in conjunction with alterations in the abundance of bacteria involved in fibre decomposition, such as Fibrobacter succinogenes and Ruminocus flavefaciens [7,20]. Overall, given that rumen fermentation is primarily influenced by rumen microbiota [21], it is predicted that the rumen microbiota was altered in this study.

Alpha diversity indices, commonly used to assess microbial richness in community ecology, are known to be negatively correlated with feed conversion efficiency [22]. In this study, Alpha diversity analyses indicated reduced bacterial diversity in the MT treatment group, which in turn suggests that cattle in this treatment group may have higher feed conversion efficiency.

Ruminal microbiota interact with hosts in a variety of ways to play a crucial role in ruminant physiology. Environmental factors, particularly dietary supplementation, can influence the composition of rumen microbial communities [23], which in turn can modulate the digestion of food, thereby influencing metabolism in ruminants [24]. Our study identified Bacteroidetes and Firmicutes as the dominant phyla represented in ruminal bacteria, which is consistent with previous studies on ruminants [25,26,27,28]. Bacteroidetes and Firmicutes are also the two most important bacteria involved in the degradation of plant polysaccharides and the production of VFAs by secreting a variety of metabolic enzymes, while other bacteria play a secondary role [29]. This may explain the similarity in dominant bacterial communities observed in ruminants that feed mainly on plant-based diets. Apart from Bacteroidetes and Firmicutes, Actinobacteria represents another key phylum, which constitutes a relatively smaller proportion of gut microbiota, but it is crucial in maintaining intestinal health and homeostasis, biodegradation of resistant starch and modulation of host immune responses [30,31,32]. Within Actinobacteria, Bifidobacteria constitute a main class of anaerobic Actinobacteria that use by glycosyl-hydrolases (GHS) to hydrolyze glycosidic bonds between two or more sugars, and cooperatively decomposes carbohydrate starch and polysaccharides, producing high concentrations of acetate that protect the host from infection of digestive tract pathogens [33]. In this study, low levels of tea saponin supplementation resulted in a significant increase in the relative abundance of Actinobacteria, which indicates that saponins have potential value in influencing livestock productivity and health by modulating ruminal bacteria.

Ruminal fungi only account for ~8% of ruminal microbiota in terms of biomass, but they are known to secrete large amounts of cellulolytic enzymes that improve the degradation and utilization of plant feed [34,35]. In this study, Ascomycota, Neocallimastigomycota and Mucoromycota were identified as the most dominant phyla in cattle’s rumen, which accords with similar findings in previous studies on Yunnan yellow cattle, gayals, yak and Tibetan yellow cattle [36]. However, a key point of difference with these other studies is that Mucoromycota was identified as a dominant phylum in this study, instead of Basidiomycota, which has been reported as the dominant phylum in other studies. Furthermore, in this study, low level supplementation with tea saponins resulted in a significant increase in the relative abundance of Ascomycota at the phylum level; and Saccharomyces and Aspergillus at the genus level. Functionally, Ascomycota are known to play a key role in degrading lignin and keratin [37]; Neocallistigomycota mainly consume rumen degradable proteins [38] and Mucoromycota are known to produce essential fatty acids and carotenoids [39]. Saccharomyces and Aspergillus play a key role in fermenting carbohydrates that are conducive to the production of TVFAs [40,41]. In addition, Saccharomyces also provides vitamins and a variety of growth factors necessary for the growth of cellulolytic-hydrolyzing bacteria [41]. Corresponding to these changes, the content of TVFAs in rumen in the low-level tea saponin treatment group was also found to be significantly increased relate to the control group in this study. Interestingly, there was no significant change in the content of TVFAs despite the significant decrease in Saccharomyces in the high-level tea saponin group, which may be related to the fact that Piromyces in the high tea saponin group compensated for the decrease in Saccharomyces. It has been previously reported that Piromyces are part of microbial consortia with high capacity of plant biomass degradation [42]. Taken together, these findings suggest that cattle in the LT supplementation treatment group had a higher ability to digest forage. In fact, we also found that the apparent digestibility of crude fibre in LT treatment group was higher. These findings are consistent with findings of previous studies that have also reported digestibility to be significantly enhanced after addition of tea saponins to forage [7].

Significant differences in microbial community structures of the four treatment groups were also identified via LEfSe analysis. More differentially abundant microbes were identified in the LT and MT treatment groups than in the CON and HT treatment groups. The LT treatment group in particular, was found to be enriched with Holdemania, Turicibacter, Propionibacterium, Akkermansia and Pseudomonas. Other researchers have reported that Propionibacterium can ferment lactate to propionate to enhance propionate production [43]. Accordingly, we also found that low level tea saponin supplementation significantly increased the proportion of propionate in rumen fermentation parameters. Enhanced propionic acid can also potentially reduce methane production in rumen and improve energy utilization efficiency [44]. Another genus that was found to be significantly enriched in the LT group was Akkermansia, which can decompose mucin and improve host health, thereby acting as a probiotics. Overall, these results indicate that tea saponins may modulate host physiology by altering the composition of ruminal microbiota.

Studies have shown that tea saponins are composed of sapogenins, glucosides and organic acids, among which glucosides include a variety of oligosaccharides [6] that are beneficial to fibre-degrading microbes [45]. Moreover, saponins can interact with cholesterol in protozoan cell membranes resulting in the destruction of protozoa [46], which then also results in reduced protozoal predation of bacteria. This could explain the increased abundance of some microbes in the low level tea saponin treatment group compared with the control group. Interestingly, the relative abundance of several microbiota such as Actinobacteria, Ascomycota, Saccharomyces and Aspergillus, were found to be lower in the high level tea saponin treatment group, compared to low level of tea saponin supplementation. Previous studies have also shown that while low level saponins can stimulate the growth of some rumen bacteria such as cellulolytic bacteria, high level saponins can inhibit the growth of microbes [47]. It has been reported that saponins can destroy the integrity of microbial cell membrane [48]. Therefore, it is possible that at high levels, tea saponins destroy the cell membrane integrity of microorganisms, thereby inhibiting their growth.

The gut microbiota is a vital regulator of host metabolism. Therefore, the functional profile of the ruminal microbial community in cattle was predicted by PICRUSt. In our study, the majority of pathways identified to be associated with ruminal microbiota were associated with metabolism, e.g., Amino acid metabolism, Metabolism of cofactors and vitamins and Carbohydrate metabolism. These results were consistent with previous studies in yaks [49] and Simmental bulls [50]. Compared with the control group, the pathways belonging to the Xenobiotics biodegradation and metabolism increased significantly in LT treatment group, indicating that low levels of tea saponins promote the metabolism of xenobiotics in cattle. Similarly, the pathways belonging to the glycolysis III increased significantly in LT treatment group, indicating that low levels of tea saponins help promote glycolysis. Several previous studies have shown plant extracts can modulate functions associated with intestinal microbiota, thereby improving host growth performance [51]. Thus, alterations in intestinal microbial function by tea saponin supplementation may lead to the performance improvements in cattle.

## 5. Conclusions

The overall aim of this study was to investigate the effect of tea saponin supplementation on ruminal bacteria and ruminal fungi of Qinchuan beef cattle via 16rRNA sequencing for the first time. Results indicate that supplementation with low levels (10 g/cattle per day) of tea saponin can significantly increase total VFAs, and the relative abundances of a variety of microbiota including Proteobacteria, Actinobacteria, Saccharomyces and Aspergillus. From a functional perspective, supplementation with tea saponins (10 g/cattle per day) was found to influence multiple metabolic pathways such as Xenobiotic biodegradation and metabolism, and glycolysis.

## Figures and Tables

**Table 1:** Ingredients and nutritional compositions of the experimental diets.

Item	Content, g/kg DM
Ingredient	
Corn silage	300
Wheat straw	100
Corn	330
Soybean meal	174
Wheat bran	66
Salt	2.4
Sodium bicarbonate	3.6
Premix ^1^	24
Nutritional level	
CP	161.9
NDF	376.9
ADF	243.7
Ca	7.5
P	4.0
ME/(MJ/kg)	10.23

DM, dry matter; NDF, neutral detergent fibre; ADF, acid detergent fibre. ^1^ The premix provided the following per kg of the diet: VA 2 560 IU, VD 3 550 IU, VE 25 mg, Fe (as ferrous sulfate) 61 mg, Cu (as copper sulfate) 18 mg, Zn (as zinc sulfate) 49 mg, Mn (as manganese sulfate) 47 mg, Co 0.12 mg, I (as potassium iodide) 0.31 mg, Se (as sodium selenite) 0.36 mg.

**Table 2:** Effects of different levels of tea saponin on rumen fermentation parameters.

Items	Treatment				SEM	*p*-Value		
	CON	LT	MT	HT		Treat	Linear	Quadratic
pH	6.31	6.27	6.26	6.25	0.04	0.13	0.07	0.37
Ammonia N, mg/100 mL	15.94	13.24	14.12	13.40	0.73	0.15	0.37	0.41
TVFAs, mmol/L	57.50 ^b^	60.17 ^a^	58.28 ^b^	57.31 ^b^	1.01	0.03	0.87	0.68
VFAs profile, %/100 mol								
Acetate	64.99	64.54	65.19	64.61	0.65	0.37	0.31	0.19
Propionate	19.68 ^b^	20.81 ^a^	20.18 ^ab^	19.63 ^b^	0.39	<0.01	0.49	0.05
Butyrate	11.31	11.35	11.50	11.69	0.02	0.87	0.04	0.88
Iso-butyrate	1.56	1.31	1.41	1.44	0.24	0.40	0.62	0.28
Valerate,	0.88	0.86	0.82	0.85	0.04	0.34	0.40	0.95
Iso-valerate	1.55	1.19	1.42	1.76	0.36	0.07	0.10	0.07
A:P	3.30 ^a^	3.11 ^b^	3.18 ^ab^	3.29 ^a^	0.13	0.02	0.86	0.01

TVFAs = total volatile fatty acid; A:P = acetate/propionate ratio. CON, tea saponins at 0 g/cattle per day; LT, tea saponins at 10 g/cattle per day; MT, tea saponins at 20 g/cattle per day; HT, tea saponins at 30 g/cattle per day. The same row with the different letters are significantly different (*p* < 0.05).

**Table 3:** Alpha diversity index of bacteria.

Items	CON	LT	MT	HT	SEM	*p*-Value
chao1 index	1423.88 ^a^	1329.05 ^ab^	1291.31 ^b^	1353.30 ^ab^	46.23	0.03
faith_pd index	75.70 ^a^	74.64 ^a^	66.25 ^b^	73.83 ^ab^	5.32	0.02
Shannon index	9.00	8.90	8.36	8.77	0.39	0.09
simpson index	0.99	0.99	0.97	0.98	0.02	0.59

CON, tea saponins at 0 g/cattle per day; LT, tea saponins at 10 g/cattle per day; MT, tea saponins at 20 g/cattle per day; HT, tea saponins at 30 g/cattle per day. The same row with the different letters are significantly different (*p* < 0.05).

**Table 4:** Alpha diversity index of fungi.

Items	CON	LT	MT	HT	SEM	*p*-Value
chao1 index	79.50	86.25	75.50	70.00	5.60	0.84
faith_pd index	7.42	7.27	7.19	5.87	0.33	0.15
Shannon index	3.85	3.71	3.39	3.47	0.09	0.39
simpson index	0.85	0.82	0.81	0.80	0.04	0.23

CON, tea saponins at 0 g/cattle per day; LT, tea saponins at 10 g/cattle per day; MT, tea saponins at 20 g/cattle per day; HT, tea saponins at 30 g/cattle per day.

**Table 5:** Species and relative abundance of ruminal fluid bacteria in cattle at phylum level and genus level.

Items	CON	LT	MT	HT	SEM	*p*-Value
Phylum level						
Bacteroidetes	68.02	66.20	67.89	69.12	2.80	0.48
Firmicutes	28.33	28.19	27.53	25.51	1.07	0.82
Proteobacteria	0.66 ^b^	1.14 ^ab^	0.91 ^b^	1.64 ^a^	0.13	<0.01
Spirochaetes	0.55	0.92	0.79	0.66	0.14	0.45
Euryarchaeota	0.53	0.75	0.73	0.27	0.17	0.49
Verrucomicrobia	0.43	0.51	0.51	0.31	0.07	0.36
Actinobacteria	0.33 ^b^	0.54 ^a^	0.23 ^b^	0.11 ^b^	0.06	0.03
Tenericutes	0.26	0.48	0.44	0.23	0.05	0.12
TM7	0.28	0.27	0.35	0.30	0.03	0.74
unclassified	0.23	0.67	0.14	0.31	0.25	0.06
Genus level						
unclassified	41.79	45.95	49.45	48.82	3.63	0.12
Prevotellaceae_Prevotella	29.64	26.59	26.58	32.04	1.19	0.40
Succiniclasticum	8.78 ^a^	6.58 ^ab^	5.84 ^b^	4.39 ^b^	0.56	0.01
Clostridium	1.35	1.54	1.30	1.85	0.16	0.35
Bacteroides	3.80 ^a^	1.24 ^b^	0.79 ^b^	1.13 ^b^	0.45	0.02
Faecalibacterium	1.85	2.29	1.07	0.51	0.32	0.62
f_Paraprevotellaceae;g_YRC22	1.06	1.04	1.50	1.77	0.16	0.15
f_Paraprevotellaceae;g-CF231	1.41	1.40	1.53	1.33	0.17	0.83
Oscillospira	0.96	1.44	1.01	0.78	0.15	0.29
Ruminococcaceae_Ruminococcus	0.63	1.26	1.03	0.59	0.18	0.24

CON, tea saponins at 0 g/cattle per day; LT, tea saponins at 10 g/cattle per day; MT, tea saponins at 20 g/cattle per day; HT, tea saponins at 30 g/cattle per day. The same row with the different letters are significantly different (*p* < 0.05).

**Table 6:** Species and relative abundance of ruminal fluid fungi in cattle at phylum level and genus level.

Items	CON	LT	MT	HT	SEM	*p*-Value
Phylum level						
Ascomycota	51.82 ^b^	75.52 ^a^	66.31 ^ab^	52.40 ^b^	3.99	0.03
Neocallimastigomycota	23.06	9.75	13.57	28.04	4.95	0.53
Mucoromycota	17.22	7.04	16.62	12.05	2.35	0.36
Cryptomycota	5.69	3.42	1.42	3.24	0.97	0.30
Basidiomycota	2.14	4.03	1.88	4.17	0.50	0.21
LKM15	0.01	0.11	0.11	0.02	0.02	0.28
unclassified	0.02	0.08	0.04	0.02	0.01	0.36
Zoopagomycota	0.02	0.04	0.02	0.05	0.01	0.12
Chytridiomycota	0.02	0.01	0.03	0.01	0.01	0.94
Genus level						
Thermomyces	29.87	36.78	31.54	32.60	2.34	0.83
unclassified	17.01	11.47	17.35	19.13	1.64	0.39
Saccharomyces	4.21 ^b^	13.27 ^a^	16.15 ^a^	2.43 ^b^	2.35	0.03
Piromyces	9.73	4.82	3.61	16.07	2.58	0.07
Rhizomucor	11.03	2.56	9.16	2.31	1.71	0.21
Aspergillus	4.02 ^b^	9.53 ^a^	4.12 ^b^	2.75 ^b^	1.02	0.04
Chaetomium	3.94 ^ab^	3.63 ^ab^	6.99 ^a^	2.11 ^b^	0.79	0.03
LKM11	5.53	2.89	1.22	3.21	0.95	0.28
Lichtheimia	2.04	0.90	1.24	4.19	0.97	0.60
Cystobasidium	1.24	2.25	1.14	3.19	0.52	0.76

CON, tea saponins at 0 g/cattle per day; LT, tea saponins at 10 g/cattle per day; MT, tea saponins at 20 g/cattle per day; HT, tea saponins at 30 g/cattle per day. The same row with the different letters are significantly different (*p* < 0.05).

## Data Availability

The raw sequence data were uploaded to the NCBI archive of sequence reads under study record number PRJNA880688 and PRJNA880642.
